# Reactivity and Pozzolanic Properties of Biomass Ashes Generated by Wheat and Soybean Straw Combustion

**DOI:** 10.3390/ma14041004

**Published:** 2021-02-20

**Authors:** Slobodan Šupić, Mirjana Malešev, Vlastimir Radonjanin, Vesna Bulatović, Tiana Milović

**Affiliations:** Department of Civil Engineering and Geodesy, Faculty of Technical Sciences, University of Novi Sad, 21000 Novi Sad, Serbia; miram@uns.ac.rs (M.M.); radonv@uns.ac.rs (V.R.); vesnam@uns.ac.rs (V.B.); tiana.milovic@uns.ac.rs (T.M.)

**Keywords:** activity index, biomass ash, cement, pozzolanic reaction, soybean, thermal analysis, wheat

## Abstract

A sustainable use of locally available wastes from agriculture as supplementary cementitious materials (SCMs) is an alternative solution for the prevention of excessive raw material usage, reduction of CO_2_ emission and cost-effective concrete production. This paper studies the reactivity of non-traditional waste SCMs: Wheat straw ash (WSA), mixture of wheat and soybean straw ash (WSSA) and soybean straw ash (SSA), which are abundant as agricultural by-products in Serbia. The chemical evaluation using XRF technique, thermal analysis (TGA/DSC), XRD and FTIR methods were performed along with physical properties tests to investigate the feasibility of utilizing biomass ashes as cement substitutes. The obtained results demonstrate a high pozzolanic activity of WSA, which is attributed to a high reactive silica content of the ash and its satisfactory level of fineness. A wider hump in XRD pattern of WSA compared to WSSA and SSA confirmed that it abounds in amorphous (reactive) phase. The insufficient activity index of soybean-based biomass ashes, characterized with a low silica content, was improved by additional grinding and/or blending with amorphous silica-rich material. This points out the mechanical activation, i.e., grinding procedure, and chemical activation, i.e., modification of the chemical composition, as techniques efficient at producing pozzolanic materials from biomass wastes. Tested biomass ashes are characterized with negligible leaching values of heavy metals, thereby satisfying eco-friendly principles of SCM utilization. The application of biomass ashes as SCMs leads to substantial cost savings, as well as benefits to the environment, such as lower consumption of cement, reduction of CO_2_ emissions during the production of cement and sustainable waste management.

## 1. Introduction

There is an urgent need to preserve natural resources and reduce CO_2_ emissions while upholding the principles of sustainable development and keeping up with the uprising concrete production. This need has prompted many researchers from around the world to explore viable options regarding these issues. A variety of experimental studies was carried out on the fresh and hardened properties and durability of pastes/mortar/concrete containing solid wastes, generated by industrial, mining, urban and agricultural activities, as a partial or full cement or aggregate replacement. Correspondingly, the construction industry has been steadily regulated to use SCMs as reflected in several standards worldwide.

The production of cement accounts for approximately 7% of the global carbon dioxide emissions, as the fabrication of one ton of ordinary Portland cement releases approximately one ton of this greenhouse gas [1]. The use of locally-available materials—industrial and agricultural waste—as engineering products, is one of the promising solutions to the economic and environmental problems of developing countries. These materials can have acceptable levels of pozzolanic activity when chemical composition, combustion conditions and level of fineness are adjusted. High silica content in the form of non-crystalline or amorphous silica and a very large surface area of material particle are the key parameters determining the possibility of application of these materials as supplementary cementitious materials (SCMs). They do not exhibit any hydraulic properties of their own; this occurs only in the presence of calcium oxide (CaO) or calcium hydroxide (Ca(OH)_2_), which are generated from the hydration process of cement. Silica and alumina in pozzolana react with these products and form more calcium silicate hydrate (C-S-H) gel, thus reducing the amount of (Ca(OH)_2_). Subsequently, concrete and mortar are stronger, denser and more durable. Since certain SCMs, like silica fume, blast furnace slag and fly ash, have already been commercialized and used in cement industry to partially replace cement, the utilization of other materials is still under investigation.

Various industry by-products, such as silica fume, blast furnace slag and fly ash are being successfully used as SCMs, thereby solving the problem of their improper disposal, conserving natural resources and reducing the amount of CO_2_ released during the production of cement clinker. Over the last few decades, a growing interest in the development of SCMs derived from industrial and agricultural wastes has been noticed in the scientific literature. The main basis for the valorization of biomass ashes lies in the fact that they contain high amounts of amorphous silica, which makes them suitable as cement substitutes in cement-based composites. Silica content of different types of biomass ashes, used as cement or small aggregate substitutes worldwide, is given in Table 1.

The focus of numerous recent studies has been on investigating the possibility of RHA, POFA, CCA and SCSA application as cement substitutes in cement-based composites, especially RHA. The optimum combustion temperature for obtaining highly reactive RHA has been determined as 600 °C [18]; the finer RHA particles exhibit better pozzolanic activity and impart higher strength [19]; at least up to 10% cement replacement with RHA results in strength development comparable to the reference cement samples [20]; blending of cement with RHA leads to a denser microstructure, lower porosity and finer pore structure, thereby inhibiting the penetration of chlorides and chemical agents and improving durability properties of cement-based composites [21].

Few researchers have provided the information on far less utilized SCMs such as wheat straw ash [4,22,23,24,25], while the materials with lower pozzolanic activity, such as oil rapeseed ash, sunflower husk ash and soybean straw ash, have been investigated to a small extent.

It is estimated that the total potential of biomass from agriculture in Serbia is about 12.5 million tons per year. The analysis of the structure of biomass from the residues of agricultural production in Serbia indicates that more than half of the resources lies in corn biomass, more than a quarter in straw of cereals, and the rest of about 15% in harvest residues of sunflower, soybean, oilseed rape or residuals from orchards and vineyards.

**Figure 1:**
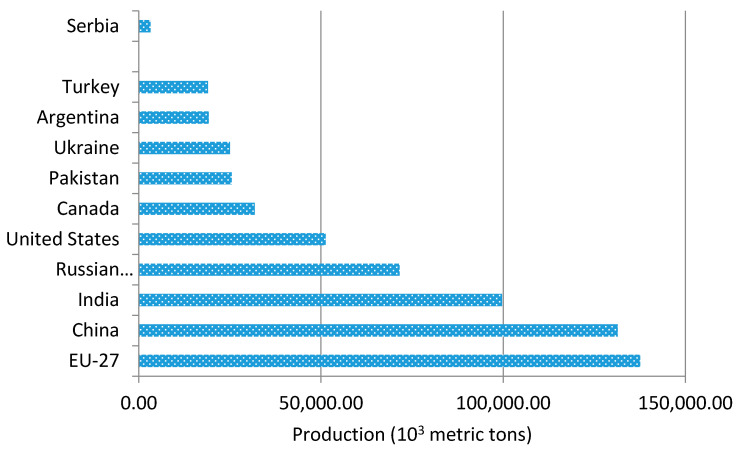
Global production of wheat, by country.

**Figure 2:**
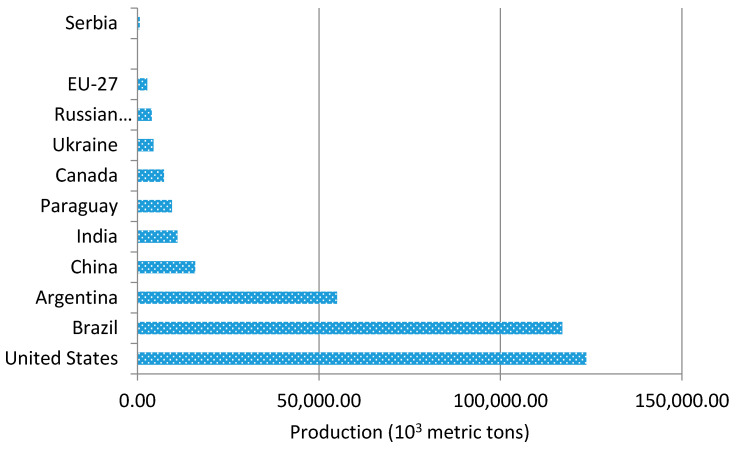
Global production of soybean seed, by country.

Global wheat utilization in 2018/19 was forecast to reach almost 743 million tons [26]. Among the leading wheat producers worldwide, Serbia is listed at number 22—Figure 1, with the annual production of 2200 metric tons [27]. Based on the soybean seed production, Serbia is ranked at number 15 [28], with the annual production of 625 metric tons—Figure 2.

It is estimated that about half of harvest residues at large agricultural farms in Vojvodina can be used for energy purposes, while only about 20% generated on relatively small private farms is utilized by the same means. The main processes generating the energy obtained from biomass include direct combustion, pyrolysis, gasification, hydro gasification, liquefaction and alcoholic fermentation. Large amounts of biomass ash are created as waste products during these processes, estimated at 5000 tons per year. They are most commonly disposed of in landfills or recycled on agricultural fields. Considering that the disposal costs of biomass ashes and biomass ash volumes are ever-increasing, a sustainable ash management has to be established.

Biomass is the largest renewable energy source in the studied region, and Serbia belongs to the top of European countries by the amount of available, but unused biomass. Furthermore, domestic industry has already produced the equipment for biomass utilization. All this suggests that there is a good prospect for a larger use of biomass, but also for generating larger quantities of ash produced by its combustion.

The use of biomass ashes as SCMs in cement composites production in Serbia have been scarcely investigated so far. Previous studies, conducted mostly on cement mortars blended with WSA [29,30], have documented that WSA blended mortar shows a promising performance in strength, depending on the level of fineness and chemical composition of the ash. However, very few studies have dealt with the microstructure and reactivity parameters of WSA as a pozzolanic material, while no studies referring to soybean straw-based ashes application can be found. In addition, locally available waste materials, originating from agriculture in Serbia, are explored and investigated for possible SCM application for the first time.

Therefore, the objectives of this study were to investigate the composition and reactivity of wheat and soybean straw-based ashes and explore the possibility of their application as pozzolanic materials in cementitious systems. Substituting cement in mortar and concrete with biomass ashes, as CO_2_ neutral fuel, would (1) reduce the negative impact of concrete industry on global warming, (2) provide a new use-value of ashes as a novel product and (3) offer the possibility of their economic valuation as such. With respect to the annually available quantities of biomass ashes in Serbia and taking into account that one ton of CO_2_ is emitted during the production of one ton of cement, a possible reduction of this pollutant in the case of managing all the generated waste in the studied region would be up to 5 million tons. All these effects would constitute a strong impetus for the creation of conditions allowing an integrated management of abundant waste materials originating from agriculture in Serbia.

## 2. Materials and Methods

### 2.1. Materials

#### 2.1.1. Cement

Ordinary Portland cement (OPC), originating from Lafarge cement factory in Beočin, Serbia, was used. The cement has a specific gravity of 3.1 g/cm^3^ and the Blaine fineness of 4.000 cm^2^/g.

#### 2.1.2. Biomass Ashes

**Figure 3:**
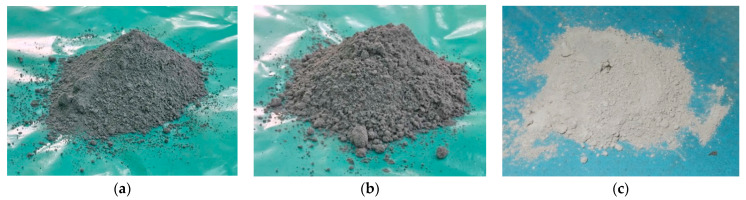
(**a**) Wheat straw ash, after sieving and grinding. (**b**) Mixture of wheat and soybean straw ash, after sieving and grinding. (**c**) Soybean straw ash, after sieving and grinding.

Samples of biomass ashes: Wheat straw ash (WSA)—Figure 3a, mixture of wheat and soybean straw ash (WSSA)—Figure 3b and soybean straw ash (SSA)—Figure 3c, were collected from three different producers in Serbia: Agricultural enterprise Mitrosrem, the soybean processing factory Sojaprotein and polypropylene factory Hipol, respectively.

The ashes were roughly sieved, through a 4 mm sieve, in order to separate un-burnt straw and other large impurities.

In order to obtain a material with high specific surface area, ashes were further ground in a laboratory ball mill until the Blaine fineness reached the range of 5500–6000 cm^2^/g (grinding time 6 h).

#### 2.1.3. Silica Fume

A commercially available product—Silica fume—was used as an additive to improve the activity index of SSA. The silica fume has a specific gravity of 2.4 g/cm^3^ and the amorphous silica content of cca 90%.

#### 2.1.4. Fine Aggregate

Standard CEN sand was used as fine aggregate for the preparation of cement pastes blended with biomass ashes.

### 2.2. Methods

Characterization of biomass ashes included several physical and chemical properties tests according to the relevant standards as well as the evaluation of the criteria conformity. The chemical composition of biomass ashes was determined using energy dispersive X-ray fluorescence spectrometer (EDXRF 2000 Oxford instruments, Belgrade, Serbia) according to EN 196-2, 2015 [31] and ISO 29581-2, 2010 [32]. The representative samples (100 g) were pulverized in a laboratory vibratory mill prior to the testing. The loss on ignition (LOI) was determined as a weight difference between 20 °C and 950 °C.

Leaching of heavy metals from biomass ashes and cement was tested via method EPA 6010C:2000 (for heavy metals: As, Ba, Cd, Co, Cr, Cu, Mn, Mo, Ni, Pb, Sb, Sn and Zn), using ICP-OES-ICPE9820, Shimadzu, Kyoto, Japan and via method US EPA 7471B:2007 (for Hg), applying AAS-(AA7000), Shimadzu, Kyoto, Japan. The preparation of test samples was carried out in accordance with EN 12457-2:2008 [33] using one stage batch test at a liquid-solid ratio of 10l/kg for materials with particle size below 4 mm (without size reduction). The instrumentation employed within the test include: Analytical balance—XT 220A Precisa, Dietikon, Switzerland, Dryer-LSW-53 Vims Electronic, Tršić, Serbia and shaker-machine for testing samples by shaking Vekamer, Novi Sad, Serbia.

Specific surface area of biomass ashes was determined according to Blaine air permeability method given in EN 196-6, 2011 [34], which is widely used for characterizing Portland cement. The test is based on the principle of resistance to air flow through a partially compacted sample of cement, and is a single parameter that is meant to identify the fineness of a powder material.

Initial and final setting time, fineness and soundness of biomass ashes were determined in accordance with EN 196-3, 2010 [35]. The method is used for assessing whether the abovementioned physical properties of a SCM material are in conformity with the requirements given in EN 450-1 [36].

The pozzolanic activity was studied on samples prepared according to the procedure given in SRPS B.C1.018, 2015 [37]. Mortars were prepared with biomass ash, slaked lime and standard sand, with the following mass proportions: m_sl_:m_bash_:m_qs_ = 1:2:9 and water—binder ratio 0.6 (where: m_sl_—mass of slaked lime; m_bash_—mass of biomass ash; m_qs_—mass of CEN standard sand). After compacting, the samples were hermetically sealed and cured for 24 h at 20 °C, then for 5 days at 55 °C. Subsequently, 24 h period was allowed for samples cooling process to reach 20 °C, followed by compressive and flexural strength tests.

The activity index of biomass ashes was examined according to EN 450-1, 2014 [36]. Activity index is defined as a ratio (in percent) of the compressive strength of standard mortar bars, prepared with 75% test cement plus 25% ash by mass, and the compressive strength of standard mortar bars prepared with 100% cement, when tested at the same age. The preparation of standard mortar bars and determination of the compressive strength were carried out in accordance with EN 196-1, 2018 [38].

Mineralogical composition was specified for all types of ashes using X-ray powder diffraction technique (XRD). XRD patterns were recorded on Philips PW1710 device, Belgrade, Serbia under the following experimental conditions: Monochromatic Cu Kα radiation with 1.5418 Å wavelength in 10–65° of 2θ range, scan rate 0.02° and 0.5 s per step, operating at 40 kV, 30 mA.

The functional groups in the ash samples were identified using Fourier Transform-Infrared Spectrometer (Thermo-Nicolet Nexus 670 FTIR spectrometer, Belgrade, Serbia). The FTIR analysis was performed under the following experimental conditions: KBr pellet technique, spectral resolution of 4 cm^−1^, range of 4000–400 cm^−1^, 32-averaged scans per one measurement.

The behavior of the ashes towards high temperatures was investigated experimentally by means of Labsys Evo (Setaram) thermal analyzer using TGA-DSC, Novi Sad, Serbia (thermogravimetric analysis-differential scanning calorimetry) as a kind of simultaneous thermal analysis-STA. TGA analysis was carried out to obtain the weight loss and DSC to obtain phase change as a function of temperature. Differentiation of the thermogravimetric data (mass loss rate) allows a better resolution and identification of weight losses (DTG). During the experiment, the ash sample was placed in an alumina crucible whereas an empty one was used as a reference. The output information from each test was a change of sample mass (TGA), mass loss rate (DTG) and heat flow (DSC), as a function of temperature. The TGA-DTG-DSC measurements were performed in the temperature range 25–1000 °C with heating rate of 10 K/min and in an argon atmosphere. The sample mass was about 40 mg.

## 3. Test Results and Discussion

### 3.1. Chemical Analyses

#### 3.1.1. Chemical Composition

The results of testing the chemical composition of biomass ashes, including the reactive silicon dioxide content, the content of chloride, soluble phosphate content and the content of free calcium oxide are summarized in Table 2.

Summarized results of chemical properties of biomass ashes, requirements in relevant standards, as well as criteria fulfillment are given in Table 3.

The pozzolanic activity of materials depends mainly on the amount of oxides: SiO_2_, Al_2_O_3_ and Fe_2_O_3_, the ratio between them and their reactivity. These compounds are responsible for improving the mechanical properties of the mix, due to the increasing development of pozzolanic reactions and formation of C-S-H products over time. Total amount of these oxides, according to EN 450-1 [36], should exceed 70%. Only WSA fulfills this requirement, while soybean-based ashes have insufficient amount of these oxides, which is expected to reflect on their pozzolanic activity.

Loss of ignition of WSA and WSSA is lower than 7% and 5%, hence these ashes fulfill the criteria for category B and A, respectively. This chemical property of SSA exceeds the limit value for all classes, which could be attributed to a higher organic content of this type of biomass ash.

The condition of passivity (high pH value) of cement-based material can be threatened due to the process of absorbing chloride ions, hence their content is limited to max 0.1%. WSA and WSSA fulfill this criterion, while SSA exceeds the indicated value.

An excessive amount of sulphates leads to an expansive effect—the formation of secondary ettringite [39], which may cause disintegration of the matrix of cement-based composite or its cracking, both leading to a reduction in overall strength of the element. All types of biomass ashes used in the research are characterized by a low amount of sulphates, satisfying the criterion defined by the given standards (≤3%).

Free lime can cause a delayed expansion and compressive strength reduction of cement-based composites, which could result in a serious deterioration of structures built [40]. All biomass ashes used in this study have low quantities of free lime.

Reactive silica is the key parameter that determines the pozzolanic potential of a material, i.e., its tendency to react with available calcium hydroxide to form cementitious hydration products. WSA has a relatively high amount of reactive silica, exceeding 67%. With the increase of soybean straw ash, silica content decreases. WSSA has a satisfactory amount, while the amount of silica in SSA is slightly below the required value.

Alkali-silica reaction (ASR) is the reaction between alkaline cement paste and amorphous silica, which is found in many common aggregates. This reaction produces swelling gel products which lead to an expansive pressure inside concrete and its cracking. In order to mitigate ASR, the alkali metal contents of the cement are limited to 5% (total alkali content). Results indicate that all types of biomass ashes are characterized by high alkali content, especially soybean-based ashes. In order to mitigate ASR, some authors suggest water treatment of raw ash as an effective way to eliminate the negative effects of high alkali content [22]. Nevertheless, non-reactive aggregates (in a crystalline form) can be used in the presence of high alkali biomass ashes.

The major effect of phosphate is to decrease alite/belite ratio, with a strong deterioration of the mechanical properties of the hydrated cement, since alite is the main hydraulic phase in clinker [41]. Phosphates, in the presence of moisture, tend to form phosphoric acid, which reduces the pH value of cement, causes corrosion of reinforcement in concrete and decelerates the disintegration of cement materials. The total phosphate content of the tested biomass ashes is within limit values, while the soluble phosphate content exceeds this figure in WSSA.

#### 3.1.2. Heavy Metals

The use of waste products as compost is subject to a common peculiarity of legislative restrictions and standards with respect to the environmental and soil conservation aspects. These restrictions play an essential role in the process of evaluating the possibility of using biomass ashes for agricultural purposes, at the same time having regard to the beneficial effects of compost. Table 4 shows heavy metal content in biomass ashes and cement and the limit values given for the use of waste materials in agriculture, in accordance with Working group Compost—Consulting and Development [42].

As follows from the figures shown above, tested biomass ashes satisfy the criteria for the use in agriculture regarding the levels of As, Cd, Cu, Ni, Pb and Zn. These concentrations are in the same range as those of cement, with the exception of Zn content, which is significantly lower in relation to the cement. The levels of Cr and Cu exceed the limit values for WSA and WSSA samples, while SSA meets the criterion for Cr level and exceeds the limit for Cu content. Therefore, the SSA sample satisfies all the requirements for its use in agriculture, with the exception of Cu content.

For the purpose of toxicity assessment of biomass ashes, in the context of these concentrations, a heavy metal index was calculated as the ratio of heavy metal content and the limit values for toxic elements. The results are given in Table 5.

The heavy metal index is an indicator of waste material quality from the aspect of its toxicity. WSA is characterized with HMI close to that of cement. With the increase of soybean ash content, total HMI increases, which can mainly be attributed to a higher concentration of Cu in soybean-based ashes.

#### 3.1.3. Leaching of Heavy Metals

Biomass ashes most commonly remain unutilized and are disposed of in a surrounding environment posing a threat to nearby ecosystems. The leaching of different heavy metals from biomass ashes could lead to a potential soil and groundwater contamination, hence the appropriate leaching test needed to be conducted. The leaching values were calculated at liquid–solid ratio L/S = 10 L/1 kg (Table 6).

These values were compared with limit values for waste materials on the landfill which are prescribed in Council Directive [43]. These limit values are given in Table 7.

Generally, all types of tested biomass ashes are characterized with no significant leaching values of heavy metals. Pursuant to the given criteria, all of them are classified as inert materials according to the content of Pb, Zn, Cu, Ni, Hg and As and non-hazardous materials based on the leaching of Cd. WSA is also classified as inert due to the content of Cr. Furthermore, with the rise of soybean straw ash content, the leaching of Cr increases. Thereby, WSSA and SSA belong to the group of non-hazardous materials.

#### 3.1.4. TGA/DSC

**Figure 4:**
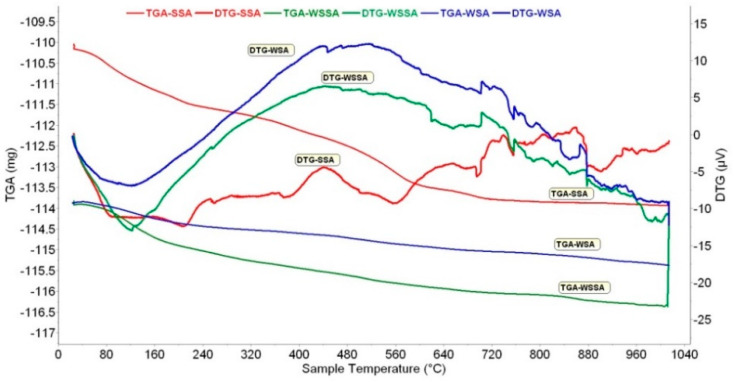
Results of TGA-DTG.

**Figure 5:**
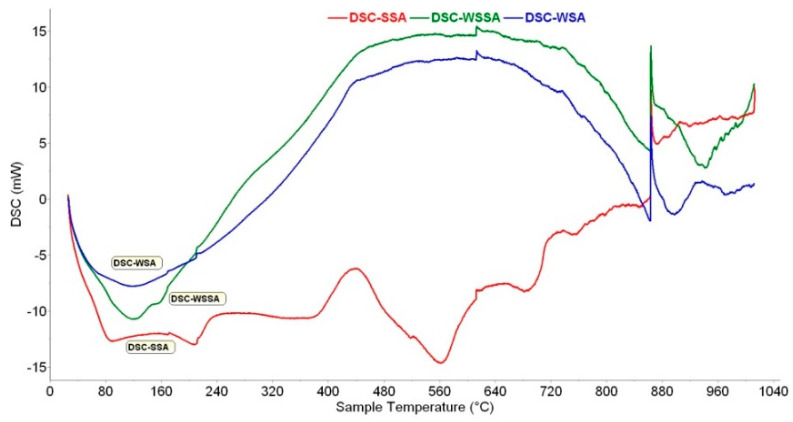
Results of DSC.

The results obtained by TGA-DTG and DSC measurements for the ashes used in the study are presented in Figure 4 and Figure 5, respectively.

Based on STA measurements, the thermal process can be divided into three stages: (1) Moisture evaporation (25–300 °C), (2) decomposition and volatilization (300–600 °C) and (3) continuous mineral decomposition (600–1000 °C).

The mass changes and broad endothermic peaks (DSC and DTG) up to 300 °C are generally assumed to be evaporation processes of physically and chemically bound water [44]. The mass loss that can be attributed to this process is: 1.60% for WSA, 3.28% for WSSA and 3.85% for SSA. These results are in good agreement with the results obtained using XRF and FTIR, confirming that SSA has the highest hygroscopicity and WSA the lowest (Table 2).

There are two likely interpretations for the occurrence of the observed exothermic peak in DSC or DTG curve between 300 and ~600 °C: (1) The volatilization of unburnt carbon and other organic matter present as a result of incomplete combustion; this was observed by Esteves at al [45], confirming the lower efficiency of the combustion process in the thermal power plant the biomass fly ash was collected at and (2) phosphorus oxide [46]. The weight loss in this range is 1.05%, 1.66% and 4.29% for WSA, WSSA and SSA, respectively. SSA has the highest value, thus explaining its highest loss on ignition given in Table 2. Moreover, the endothermic peak, visible only in SSA, centered at about 560 °C, may correspond with calcium carbonate decomposition [47].

In the next range 600–850 °C, the observed endothermic peaks probably correspond to the decomposition of carbonates and release of CO_2_ [44,48]. The mass loss in this interval is 0.61% for WSA, 0.71% for WSSA and 1.04% for SSA. The presence of increasing amounts of alkali components can decrease the decomposition temperature of CaCO_3_ [47], like in the case of SSA.

The mass losses observed at higher temperatures are mainly attributed to the evaporation of alkali-containing components. Therefore, the reduction in mass noted in the temperature segment 850–1000 °C characterized by the endothermic DSC peak (especially in SSA) is likely to be assigned to KCl evaporation, K_2_CO_3_ decomposition [44] or formation of SO_2_ [49]—reduction of sulfates or melting.

DTG curves of WSA and WSSA start bending down rapidly after ~700 °C, showing the initiation of a melting effect among the ash constituents [50].

The total mass losses from room temperature to 1000 °C are: 3.77% for WSA, 6.13% for WSSA and 9.35% for SSA. The mass losses of tested ashes at different temperature intervals are given in Table 8. These percentages were calculated with respect to the initial mass of samples.

A discrepancy in weight loss results obtained using LOI and TGA could be attributed to an incomplete decomposition of all species.

#### 3.1.5. FTIR

**Figure 6:**
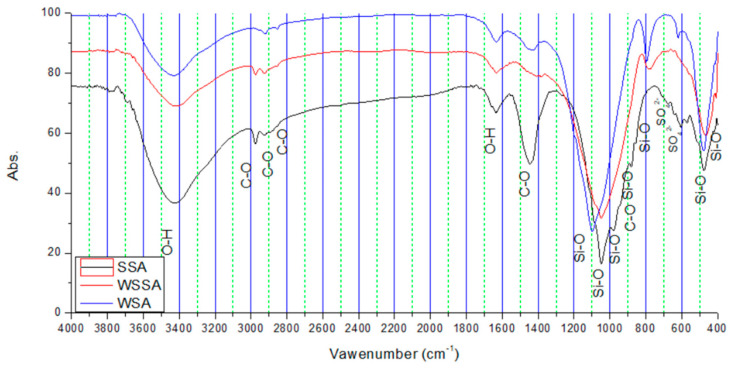
FTIR spectra of tested biomass ashes.

FTIR spectrograph shown in Figure 6 was observed in order to identify functional groups present in the tested ashes. The broad peak at about 3400 cm^−1^ (characteristic of all three samples) is allocated to the OH-stretching vibration of adsorbed water bands whereas the one at 1630 cm^−1^ is associated to the OH-bending vibration in H_2_O molecules [51]. Also, several peaks in the range 3400–4000 cm^−1^ may be assigned to absorbed water. These peaks are the most intense for SSA, which coincides with the results obtained by other analyses.

The peaks appearing in the region 2920–2925 cm^−1^ in all the samples show the presence of organic carbon [52]. These peaks are particularly pronounced in SSA.

A spectral band between 1380 and 1450 cm^−1^ is characteristic of the asymmetric stretching vibration of C=0 group, which suggests the presence of carbonate compounds in the samples [53,54]. The band at 1447 cm^−1^, distinctive in SSA, has the highest intensity and it is typical for alkaline carbonates [54].

The most intense band observed for all ashes is at approximately 1000–1100 cm^−1^, and is attributed to the asymmetric stretching vibrations of Si(Al)–O in silica and aluminosilicate phases [55,56]. This band provides information on the degree of crystallinity/amorphicity of a sample [57]. The interpretation of the FTIR spectra in this range may be very difficult because the bond vibrations tend to overlap. The broad band in this range, which is the most intensive in the WSA sample, is suggested to be related to an amorphous aluminosilicate phase [58]. Al/Si ratio increases with the decreasing wavenumber values, as in the case of WSSA and SSA in relation to WSA.

The band detected at 979.96 cm^−1^ in SSA is mainly associated with the stretching vibration of Si-O-Si(Al) [59,60] while the peak appearing at around 880 cm^−1^, corresponds to CO^3−^ ion (carbonated ion, probably potassium carbonate) [61,62,63,64].

The band at 794.86 cm^−1^ which is visible in WSA and the band at 776.81 cm^−1^ visible in WSSA are due to the Si-O-Si stretching vibration (probably cristobalite and quartz) [64,65,66]. This band is not detected in SSA. Also, the band situated at 620 cm^−1^ in WSA signifies the presence of cristobalite phase, which was also observed in the biomass ash examined by Osman et al. [67]. The presence of calcium sulfate in the form of anhydrite may be confirmed by the vibration band appearing at 604 cm^−1^, typically found in SSA [57].

The band at 570.61 cm^−1^ visible only in SSA comes from Al-O-SI vibration (mullite), as noted by Cretescu et al. [64,68].

The band values under 500 cm^−1^ characteristic for all samples indicate the presence of the Si-O functional group [69]. The peaks near 466 cm^−1^ in WSSA correspond probably to a mode of feldspar [52].

#### 3.1.6. X-ray Diffraction

**Figure 7:**
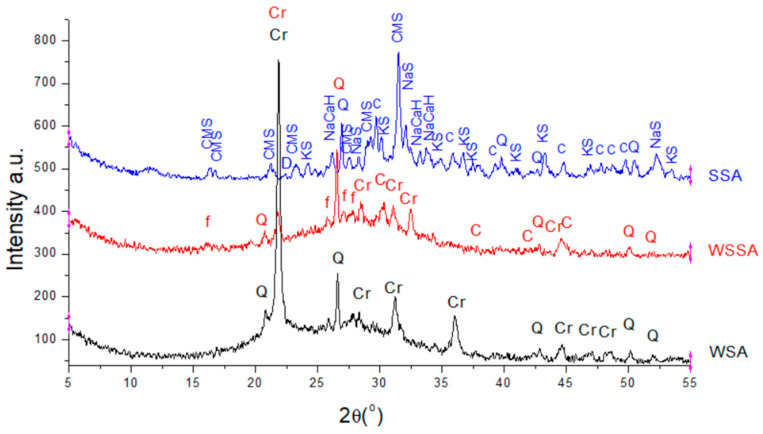
XRD pattern of biomass ashes (Q-quartz, Cr-cristobalite, f-feldspar-like minerals, C-calcite, CMS-calcium magnesium silicate, NaS-sodium sulphate, KS-potasium sulphate, NaCaH-sodium calcium hydrogen, D-dolomite).

The X-ray diffractogram of tested biomass ashes is shown in Figure 7 and it was taken to determine the crystalline phases present in the ashes. WSA comprises amorphous phases (major part) as indicated by a characteristic broad hump from 17° to 35° (2θ). The main crystalline components of this ash are cristobalite and quartz (both are SiO_2_). The peaks of cristobalite are the most intensive. A similar result was obtained for WSSA but with the hump from 20° to 35° (2θ) and a lower intensity of peaks corresponding to cristobalite and quartz. Cristobalite in amorphous form is also specific for wheat husk ash examined by Hernández-Martínez et al. [66]. Feldspar and calcite were also observed in WSSA.

The intensity of phase diffraction peaks is proportional to the concentration of components influencing it; likewise, the difference in peak phase intensities symbolizes the difference in the concentration of phase constituents. It is known that amorphous silica, being more reactive than crystalline silica, is preferred for pozzolanic reaction [70]. The high silica content in amorphous state (especially in WSA) gives ashes the potential for use as a pozzolanic material in cement-based mixtures.

The XRD pattern of SSA shows a large number of small diffraction peaks: Predominantly silicate, related to quartz, sulphate in a form of sodium and potassium sulphate, magnesium as dolomite and calcium magnesium silicate as well as carbonate associated with calcite and dolomite. According to the size of hump, this type of biomass ashes has fewer amorphous phases centered at about 32° (2θ) than WSA and WSSA.

### 3.2. Physical Properties of Biomass Ashes

Physical properties of tested biomass ashes are shown in Table 9.

All types of ashes have similar specific gravities, cca 2400 kg/m^3^. After grinding in a laboratory ball mill for 6 h, specific surface area (Blaine) of biomass ashes reached 5500 cm^2^/g.

### 3.3. Pozzolanic Activity

Pozzolanic material class was determined based on 7-day compressive (f_c_) and flexural (f_f_) strength of standard mortar prisms. Results revealing the pozzolanic properties of tested biomass ashes are given in Table 10.

As can be seen from the figures shown above, WSA displays a pozzolanic activity of Class 10, WSSA demonstrates a pozzolanic activity of Class 5, while SSA, manifests an insufficient activity to achieve this type of class. The achieved class of pozzolanic activity of biomass ashes, as potential pozzolanic materials, primarily depends on the amount of reactive silica. As the amount of soybean straw increases, reactive silica content is reduced and the class of pozzolanic activity decreases. This is a direct consequence of a lower amount of reactive silica in soybean-based biomass ashes, as discussed in previous chapters.

### 3.4. Setting Time

The initial setting time, as specified in EN 197-1 [70], shall not be more than twice as long as the initial setting time of a 100% (by mass) reference cement paste (criterion 1). The initial setting time, should not be shorter than 60 minutes—criterion 2 (for cement type CEM I 42,5R). All types of tested biomass ashes fulfill these criteria. The results are given in Table 11.

The addition of WSA did not retard the initial setting time, as expected when it comes to SCMs; instead, the presence of this ash slightly accelerated it. As the hydration process of a pozzolanic material takes longer time and retards the setting time, the final setting time of WSA was extended.

The overall effect of soybean-based biomass ashes on the setting time of cement paste was proven to retard the setting time. Both initial and final setting time were extended in relation to the setting time of OPC. WSSA has a considerably increased initial setting time, which may be caused by the presence of unburnt organic substances—straw remains.

An extended setting time could be an advantage, as it makes cement composites remain workable for a longer period of time, which is especially useful in warmer days.

### 3.5. Soundness

According to the criteria given in EN 196-3 [35] and EN 197-1 [70], the soundness shall not be greater than 10 mm. As all types of biomass ashes showed negligible expansion, up to 1 mm, the criteria are fulfilled. The results are given in Table 12.

### 3.6. Activity Index

**Figure 8:**
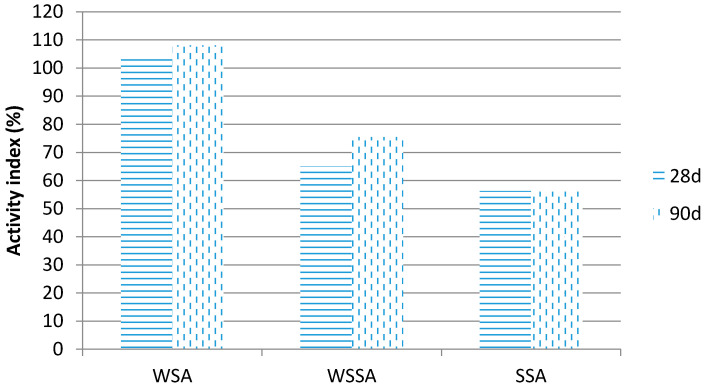
Activity index of biomass ashes.

The results of testing activity index are presented in Figure 8.

According to the criteria given in standard EN 450-1 [36], the activity index at 28 days and at 90 days shall not be less than 75% and 85%, respectively. The WSSA and SSA samples do not meet these requirements, while WSA fulfilled not only the prescribed criteria but it achieved compressive strength higher than that of the reference mortar. At the age of 28 days, the pozzolanic reaction of WSA was not intensified yet and thus the strength increase can be attributed to the filler effect of small particles of WSA.

Other tested ashes, WSSA and SSA, do not meet standards due to a lower amount of reactive silica and, consequently, insufficient pozzolanic activity.

### 3.7. Improving Activity Index with Additional Grinding

**Figure 9:**
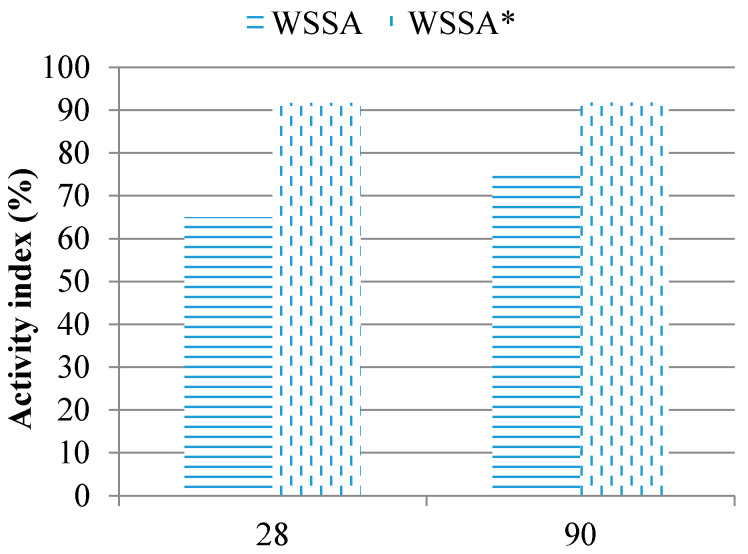
Activity index of additionally ground WSSA (* additionally ground sample).

**Figure 10:**
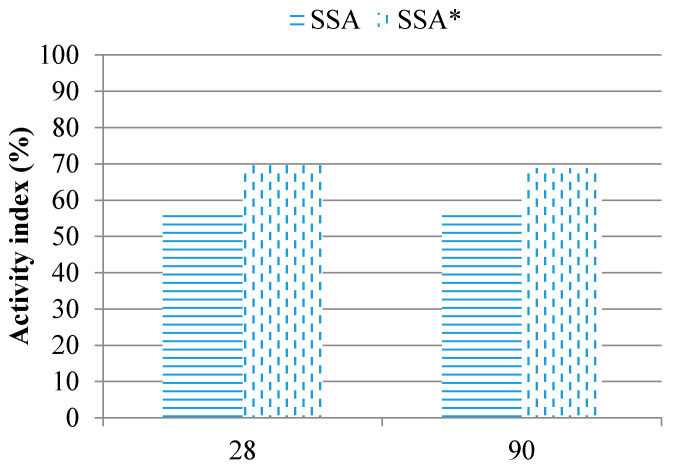
Activity index of additionally ground SSA (* additionally ground sample).

One of the key factors affecting the reactivity of pozzolans is their fineness. In order to improve filler effects of the biomass ashes with insufficient activity index, the ashes were additionally ground (for 12 h), achieving thereby Blaine fineness of 15,000.00 cm^2^/g. This approach enabled recording the pure effect of fineness on biomass ash reactivity (filler effect). The results are presented in Figure 9 and Figure 10.

Additional grinding increased the level of fineness of biomass ashes, which had a positive impact on their activity index.

The activity index of WSSA, after 28 and 90 days, was 91.7% and 91.8%, respectively, thereby exceeding the required values. At the age of 28 days, the activity index was increased by 27% (from 65 to 91.7%). At the age of 90 days, there was a rise in the activity index by 18% (from 75.5% to 91.8%).

A growth in SSA activity index of 19% (from 56.3% to 69.9%) was detected at the age of 28 days, along with an increase of 19% (from 56% to 68.8%) at the age of 90 days. The achieved values were still below the required criteria, which is a direct consequence of low reactive silica content of this type of biomass ash.

### 3.8. Stimulation of Pozzolanic Activity of SSA with Silica Fume

Two main factors influencing a pozzolanic activity are a reactive silica content and a level of fineness. As the raised level of fineness did not improve the activity index of SSA enough, silica fume, which contains a large amount of reactive silica (90%), was used to stimulate its pozzolanic reaction.

**Figure 11:**
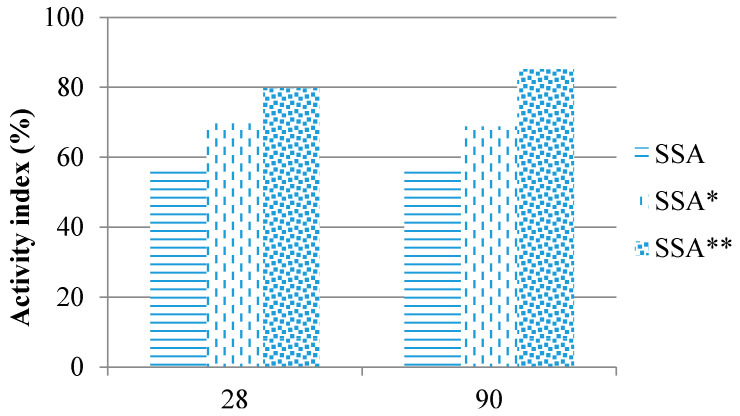
Activity index of additionally ground SSA (* additionally ground sample, ** sample blended with silica fume).

The quantity of silica fume was determined stochastically. The replacement level of cement with the mixture of biomass ash and silica fume (in the mass ratio 2:1) was kept at 25%. The aim was to increase the amount of reactive silica of soybean-based biomass ash, which would influence its pozzolanic activity and improve the activity index (Figure 11).

The criteria regarding the activity index of soybean straw ash blended with silica fume were satisfied. The activity index was 80% and 85.2% at the age of 28 and 90 days, respectively.

Biomass ash reactivity depends essentially on its fineness, vitreous phase content and chemical composition (reactive silica). Modifying the first parameter via mechanical activation and others via chemical activation are the possible routes to heighten and control their reactivity. The results are in line with Jiménez’s research on the reactivity of fly ash [71]. Moreover, the grinding time affects the final pozzolanic performance remarkably, as higher surface area values of the ash lead to more reactive sites for a pozzolanic reaction. Similar trend was observed by Thiedeitz et al. [72].

## 4. Conclusions

The aim of this research was to analyze the ashes originating from harvest residues—WSA, WSSA and SSA—locally available in Vojvodina province. These waste materials are generated at large and small agricultural farms in Vojvodina, derived as by-products within biomass combustion for the purpose of heat energy production. More specifically, the task was to investigate their potential as sustainable building materials by determining their chemical and physical properties and pozzolanic activity. Biomass ash reactivity depends heavily on its fineness, amorphous phase content and chemical composition (reactive silica content). Modifying these parameters via mechanical and/or chemical activation could contribute to a greater reactivity of these waste materials. Based on the conducted survey, the following conclusions can be drawn:

- WSA exhibits excellent pozzolanic activity, which can be attributed to a high reactive silica content and a relatively high specific surface of the ash. As the content of soybean-based ash increases, the total reactive silica content decreases, which further reduces the pozzolanic activity of the ash;
- SSA shows the highest hygroscopicity as confirmed by XRF, TGA and FTIR analyses; it also contains the largest amount of carbonates and organic matter relative to WSA and WSSA (confirmed by TGA, FTIR and XRD);
- A wider hump in XRD pattern of WSA and WSSA compared to SSA indicates that they abound in amorphous phase (silicate) which accounts for their higher pozzolanic activity to a certain extent;
- Additional grinding in the laboratory ball mill increases the level of fineness of WSSA, induces its filler effect and improves its activity index. Mechanical activation has only a minor effect on SSA reactivity, hence silica fume can be used to stimulate its pozzolanic activity;
- All types of biomass ashes are characterized with no significant leaching values of heavy metals and can be classified as inert materials, thereby satisfying ecological aspects of SCM utilization.

It can be stated that mechanical activation through grinding procedure and chemical activation by modifying the chemical composition of the ash are efficient at producing pozzolanic materials from biomass wastes. Also, the choice of activation method depends on the chemical composition of biomass ash, i.e., its reactive silica content. The presented research shows that non-traditional waste materials, specifically soy-bean straw ashes characterized by low silica content, can be exploited as potential cement substitutes when some of the proposed adjustment/processing techniques are used. An environmentally friendly route for this type of waste was defined, offering an opportunity for the creation of new sustainable cement-based composites.

## Figures and Tables

**Table 1:** Silica content of various biomass ashes.

	Silica Content (%)	Reference
Rice husk ash (RHA)	88.0	Ki-Bong et al. [2]
Corn cob ash (CCA)	61.8	Shazim et al. [3]
Wheat straw ash (WSA)	65.7	Qudoos et al. [4]
Palm oil fuel ash (POFA)	63.6	Chindaprasirt et al. [5]
Sugar cane straw ash (SCSA)	36.5	Moraes J.C.B. et al. [6]
Sunflower stalk ash (SSA)	26.0	Aksog˘an O. et al. [7]
Bamboo leaf ash (BLA)	80.4	Cociñaa E.V. et al. [8]
Groundnut shell ash (GSA)	41.4	Alaneme K.K. et al. [9]
Saw dust ash (SDA)	69.3	Raheem A.A. et al. [10]
Oyster shell ash (OSA)	4.6	Gengying Li et al. [11]
Mischantus ash (MA)	57.0	Wigley F. et al. [12]
Barley ash (BA)	31.0	Risnes H. et al. [13]
Sunflower husk ash (SHA)	29.3	Demirbas A. et al. [14]
Olive husk ash (OHA)	32.7	Demirbas A. et al. [14]
Coconut shell ash (CSA)	66.3	Opeyemi J. et al. [15]
Rape straw ash (RSA)	36.7	Masiá T.A.A. et al. [16]
Eucalyptus biomass ash (EBA)	1.2	Teixeira A.H.C. [17]

**Table 2:** Chemical composition of biomass ashes.

	Wheat Straw Ash (WSA)	Mixture of Wheat and Soybean Straw Ash (WSSA)	Soybean Straw Ash (SSA)
Loss of ignition at 950 °C	5.18	4.85	10.73
Moisture loss at 105 °C	0.95	1.00	3.62
SiO_2_, %	69.13	56.36	32.62
Al_2_O_3_, %	1.12	2.03	4.58
Fe_2_O_3_, %	0.73	1.53	1.46
Na_2_O, %	0.11	0.20	0.85
K_2_O, %	13.03	20.02	20.96
MgO, %	2.5	3.54	8.33
CaO, %	5.78	7.13	15.78
SO_3_, %	0.2	0.18	0.47
P_2_O_5_, %	1.72	3.72	3.72
Soluble P_2_O_5_, mg/kg	13.77	115.95	<0.05
Content Cl^−^, %	0.060	0.025	0.127
Free CaO content, %	0.14	0.28	0.39
Reactive SiO_2_ content, %	67.07	40.99	24.93

**Table 3:** Chemical composition of biomass ashes—criteria fulfillment.

	Chemical Requirements (EN 450-1)	Criteria	Standard	WSA	WSSA	SSA
**Chemical Properties**	Total amount of oxides: SiO_2_ + Al_2_O_3_ + Fe_2_O_3_ (%)	≥70%	EN 196-2 EN 450-1	71.0	57.12	39.04
	Loss of ignition (%)	A: Max 5% B: Max 7% C: Max 9%	EN 196-2 EN 450-1	5.1 CLASS B	4.85 CLASS A	10.93
	Chloride content (%)	≤0.1%	EN 196-2 EN 450-1	0.060	0.025	0.127
	Sulphate content (%)	≤3%	EN 196-2 EN 450-1	0.2	0.18	0.47
	Free CaO content (%)	≤1.5%	EN451-1 EN 450-1	0.14	0.28	0.39
	Reactive SiO_2_ content (%)	≥25%	EN197-1 EN 450-1	67.07	40.99	24.93
	Total amount of alkalis (%) Na_2_O+0.658 K_2_O	≤5%	EN 196-2 EN 450-1	8.68	13.37	14.64
	Phosphate content (%)	≤5%	ISO 29581-2 EN 450-1	1.72	3.72	3.72
	Soluble phosphate content (mg/kg)	≤100 mg/kg	EN 450-1	13.8	116	0.05

**Table 4:** Heavy metal content in the tested biomass ashes, cement and the limit values given for the use of waste materials in agriculture.

	WSA	WSSA	SSA	Cement	Limit Value
As, mg/kg	0.03	0.03	0.03	0.03	200
Ba, mg/kg	407.00	325.01	225.01	263	-
Cd, mg/kg	0.03	0.03	0.03	0.03	0.7
Co, mg/kg	0.01	0.01	0.01	0.01	-
Cr, mg/kg	71.25	87.25	67.50	97	70
Cu, mg/kg	84.25	107.75	172.52	0.01	70
Hg, mg/kg	0.01	0.01	0.01	0.01	0,4
Mn, mg/kg	500.00	587.01	335.12	470	-
Mo, mg/kg	0.01	0.01	0.01	0.01	-
Ni, mg/kg	0.01	0.01	0.01	0.01	25
Pb, mg/kg	0.01	0.01	0.01	0.01	45
Sb, mg/kg	0.03	12.50	335.01	0.05	-
Sn, mg/kg	0.05	0.05	0.05	0.05	-
Zn, mg/kg	5.00	37.50	51.25	150	200

**Table 5:** Heavy metal index—HMI for the use of waste materials in agriculture.

	WSA	WSSA	SSA	Cement
As, mg/kg	0.00	0.00	0.00	0.00
Cd, mg/kg	0.00	0.00	0.00	0.00
Cr, mg/kg	1.02	1.25	0.96	1.39
Cu, mg/kg	1.20	1.54	2.51	0.00
Hg, mg/kg	0.00	0.00	0.00	0.00
Ni, mg/kg	0.00	0.00	0.00	0.00
Pb, mg/kg	0.00	0.00	0.00	0.00
Zn, mg/kg	0.025	0.187	0.26	0.75
HMI sum	2.245	2.98	3.73	2.14

**Table 6:** Leaching of heavy metals from biomass ashes in mg/kg ds (dry substance).

	Pb	Cd	Zn	Cu	Ni	Cr	Hg	As
WSA	<0.07	0.27	<0.4	0.36	<0.1	0.44	<0.003	<0.15
WSSA	<0.07	0.22	<0.4	1.5	0.13	0.62	0.01	<0.15
SSA	<0.07	0.25	<0.4	0.84	<0.1	1.70	<0.003	<0.15

**Table 7:** Leaching limit values of heavy metals, in mg/kg ds.

	Pb	Cd	Zn	Cu	Ni	Cr	Hg	As
Inert	0.5	0.04	4	2	0.4	0.5	0.01	0.5
Non-Hazardous	10	1	50	50	10	10	0.2	2
Hazardous	50	5	200	100	40	70	2	25

**Table 8:** The mass losses (%) of ash at different temperature intervals.

Biomass Ash/Temperature	25–300 °C	300–600 °C	600–850 °C	850–1000 °C	Total
WSA	1.6	1.05	0.61	0.51	3.77
WSSA	3.28	1.66	0.71	0.48	6.13
SSSA	3.85	4.29	1.04	0.17	9.35

**Table 9:** Physical properties of tested biomass ashes.

	Specific Gravity (kg/m^3^)	Specific Surface Area (Blaine) (cm^2^/g)
WSA	2380	5800
WSSA	2370	5500
SSA	2400	5600

**Table 10:** Pozzolanic activity of tested biomass ashes.

Biomass Ash	f_f,av_ (MP)]	f_f,min_ (MPa)	Flexural Strength Class	f_c,av_ (MPa)	f_c,min_ (MPa)	Compressive Strength Class	Class
WSA	3.4	3.3	10	11.0	10.3	10	10
WSSA	3.6	3.3	10	9.3	8.75	5	5
SSA	1.6	1.45	/	4.06	3.84	/	/

f_f,av_—average flexural strength; f_f,min_—minimal flexural strength; f_c,av_—average compressive strength; f_c,min_—minimal compressive strength.

**Table 11:** Initial and final setting time of biomass ashes.

	The Initial Setting Time (Minutes)	The Final Setting Time (Minutes)	Criterion 1	Criterion 2
C	230	275	fulfilled	fulfilled
WSA	220	320	fulfilled	fulfilled
WSSA	350	420	fulfilled	fulfilled
SSA	310	355	fulfilled	fulfilled

**Table 12:** Soundness of biomass ashes.

	Expansion (mm)	Criterion
C	0	fulfilled
WSA	0.5	fulfilled
WSSA	1	fulfilled
SSA	1	fulfilled

## Data Availability

Data sharing not applicable.
